# Effects of Information Load on Schema and Episodic Memory Formation

**DOI:** 10.3389/fnbeh.2022.923713

**Published:** 2022-07-11

**Authors:** Maximilian Harkotte, María P. Contreras, Marion Inostroza, Jan Born

**Affiliations:** ^1^Institute of Medical Psychology and Behavioral Neurobiology, University of Tübingen, Tübingen, Germany; ^2^Graduate Training Centre of Neuroscience, International Max Planck Research School, Tübingen, Germany; ^3^German Center for Diabetes Research (DZD), Institute for Diabetes Research and Metabolic Diseases of the Helmholtz Center Munich at the University of Tübingen (IDM), Tübingen, Germany; ^4^Center for Integrative Neuroscience, University of Tübingen, Tübingen, Germany

**Keywords:** schema memory, episodic memory, information load, object recognition, memory tradeoff

## Abstract

The formation of semantic memories is assumed to result from the abstraction of general, schema-like knowledge across multiple experiences, while at the same time, episodic details from individual experiences are forgotten. Against this backdrop, our study examined the effects of information load (high vs. low) during encoding on the formation of episodic and schema memory using an elaborated version of an object-place recognition (OPR) task in rats. The task allowed for the abstraction of a spatial rule across four (low information load) or eight (high information load) encoding episodes (spaced apart by a 20 min interval) in which the rats could freely explore two objects in an open field arena. After this encoding phase, animals were left undisturbed for 24 h and then tested either for the expression of schema memory, i.e., for the spatial rule, or memory for an individual encoding episode. Rats in the high information load condition exhibited a more robust schema memory for the spatial rule than in the low information load condition. In contrast, rats in the low load condition showed more robust memory for individual learning episodes than in the high information load condition. Our findings of opposing effects might point to an information-load-dependent competitive relationship between processes of schema and episodic memory formation, although other explanations are possible.

## Introduction

Forming new memories through learning is contingent on prior knowledge (Winocur et al., 2010; Ghosh and Gilboa, 2014; Gilboa and Marlatte, 2017). For example, understanding the content of a scientific article relies on concepts or mental schemas that the reader already possesses. More formally, mental schemas can be described as higher-level knowledge structures that organize lower-level representations in long-term memory (Gilboa and Marlatte, 2017; Klinzing et al., 2019). Schemas can take different shapes, such as narratives about causal relationships, concepts, and categories in which we understand the world or knowledge about recurrent patterns. It is assumed that the formation of schema memory relies on the abstraction of experiences from single or multiple episodes into general, more abstract knowledge that lacks episodic detail also referred to as event gist (Inostroza and Born, 2013; Gilboa and Marlatte, 2017; Sekeres et al., 2018; Alonso et al., 2020). The timescale over which such abstraction occurs differs for the type of schema memory under investigation. For instance, narratives about causal relationships can be encoded within a single episode, whereas the often implicit knowledge about recurring patterns forms over multiple episodes (Tse et al., 2007; Gilboa and Marlatte, 2017; Genzel et al., 2019). Multiple experiences generally benefit the formation of schemas, either through the assimilation of new external information into mental schemas or the adaptation of already existing schemas by taking into account new related information (Winocur et al., 2010; Gilboa and Marlatte, 2017).

In contrast to schema memory, in episodic memory, the details of a single episode are retained with high fidelity. Systems memory consolidation processes may both strengthen episodic memories and promote the transition from episodic to schema memory (Inostroza and Born, 2013; Schapiro et al., 2017). Episodic details are lost either through processes of decay or processes of interference (Sadeh et al., 2014, 2016; Polack et al., 2017; Sun et al., 2017), and recent studies in humans demonstrated that schema memory guides the recall of spatial and item memories of an experienced episode after longer retention times when their relative strengths change (Zeng et al., 2021; Ramey et al., 2022). However, it is unknown whether these processes can occur in parallel or are competing processes as they rely on similar neural structures. To date, only a few studies have examined this question. Evidence from human studies suggests that a vast amount of information during learning impedes the formation of persisting episodic memory representations (Feld et al., 2016; Feld and Born, 2017; Kolibius et al., 2021). Indeed, assuming that sleep is necessary for consolidating episodic memory, Kolibius et al. (2021) proposed that the effect of memory consolidation scales with information load due to a limited capacity available for sleep-dependent memory consolidation. Therefore, information load at learning might mediate the formation of schema and episodic memory in opposite directions, i.e., schema memory benefits from larger amounts of information whereas episodic memory becomes blurred.

Here, we aimed to test this hypothesis in adult rats using an elaborated version of the object-place recognition (OPR) task. Although many paradigms have been established to study different aspects (i.e., what, where, and when components) of episodic memory in rodents (Binder et al., 2012; Takeuchi et al., 2016; Oyanedel et al., 2019), there are only a few attempts to study the behavioral expression of schema memory. For example, using an object-place reward learning task, McKenzie et al. (2014) demonstrated that such object-place associations are hierarchically represented in hippocampal structures, presumably supporting processes of pattern separation and completion in schema memories. However, a caveat of these and similar tasks (Tse et al., 2007) is the use of emotional stimuli, positive rewards, or aversive electrical shocks, that may bias the formation of schema memory. Additionally, these tasks often do not allow for an assessment of truly episodic memory (i.e., for an event occurring in a unique spatio-temporal context) because the animals need to be trained repetitively with the same task stimuli. Hence, for contrasting the formation of schema memory and episodic memory in an unbiased manner, tasks like the OPR task might be advantageous as they exploit the rodents’ natural tendency to explore novelty (Binder et al., 2012; Oyanedel et al., 2019). Findings that rats and mice are able to form a cumulative memory for a spatial rule in an adapted version of the OPR task (Genzel et al., 2019) represent the first evidence that such tasks provide a promising approach to the joint assessment of episodic and schema memory in rodents.

Accordingly, here, we used an elaborated version of the OPR task to examine the question of whether information load during learning affects the formation of episodic and schematic memory in opposite directions. The task consisted of either four (low information load) or eight (high information load) consecutive encoding episodes, in which animals explored different pairs of identical objects. To test schema memory, the objects were positioned according to a spatial rule across all episodes, and memory was assessed 24 h later by positioning the objects such that one object violated the spatial rule. Based on the rodent’s natural tendency to explore novelty, we expected animals that had successfully formed a schema memory for the rule, to preferentially explore the object that violated the rule. To test episodic memory, we presented the rats also with four or eight encoding episodes, but with no spatial rule present across episodes. Memory was assessed for the last encoding episode again 24 h later, by re-exposing the animal to these objects with one object displaced to a different location. Rats that successfully formed an episodic memory were expected to preferentially explore the displaced object. We hypothesized that high information load during encoding supports schema memory formations while episodic memory is absent. Conversely, a low load of information during encoding should result in episodic memory but not schema memory.

## Material and Methods

### Animals

Forty adult male Long-Evans rats (Janvier, Le Genest-Saint-Isle, France), 9–12 weeks old at the beginning of the experiment, were used in this study. Rats were housed in groups of two-four per cage with ad libitum access to food and water throughout the experiment and were kept on a 12 h/12 h light-dark cycle (lights on at 6:00 a.m.). Before starting behavioral testing, animals were handled daily for 10–15 min on five consecutive days. All experimental procedures were performed in accordance with the European animal protection laws and policies and were approved by the Baden-Wuerttemberg state authorities.

### Apparatus and Objects

An elaborated version of the object-place recognition (OPR) task was performed in a quadratic open field arena (80 × 80 × 40 cm, made of gray PVC), which was dimly lit with 20–30 lux and equipped with a masking white noise of 60 dB. A camera (Logitech C920) was mounted above the open field. The camera as well as posters affixed to the walls of the testing room and surrounding curtains represented distal spatial cues. Eight pairs of glass objects of different shapes and sizes (height 15–30 cm, bottom diameter 7–12 cm), filled with sand of different colors, were used in the experiments. To assure that rats could effectively discriminate the different objects, only sets of objects were used that had previously been tested in experiments that used the novel object recognition task (Sawangjit et al., 2018). Objects had sufficient weight to ensure that rats could not move them. The arena and objects were cleaned after each trial with 70% ethanol solution to prevent variance in smell.

### Experimental Procedures, Task, and Design

Prior to the experiments, animals were habituated to the testing room and open field arena. For that, animals were brought inside their home cage into the testing room on three consecutive days before the experiment was performed. After the animals spent at least 15 min inside the testing room they were placed inside the empty open field arena facing a different wall of the arena during each habituation session. The animals could then freely explore the arena and its surrounding cues for 10 min. Afterward, animals were brought back to their home cage and to the animal facility where they were kept.

**Figure 1:**
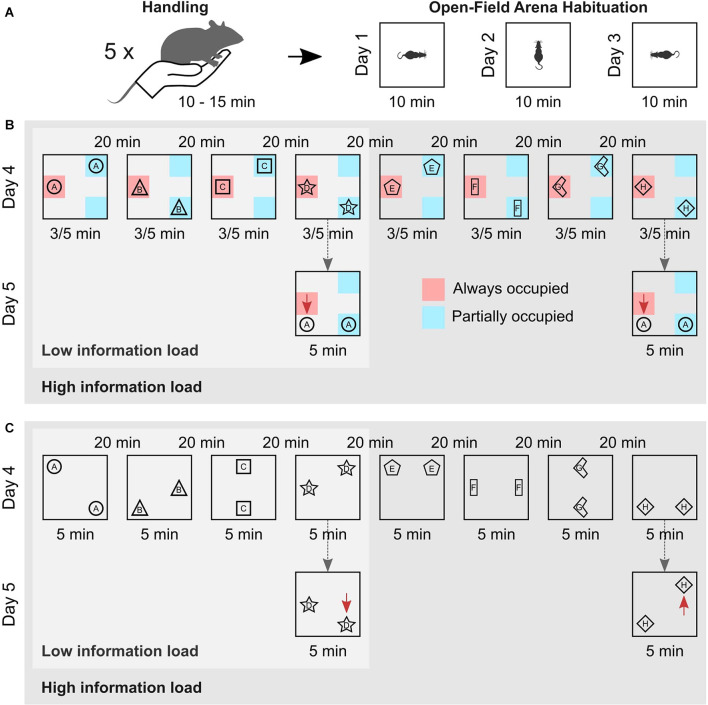
Experimental procedure. **(A)** Animals were handled on five consecutive days for 10–15 min prior to the experiment (left). Then, they were habituated to the empty arena for 10 min on three consecutive days. They entered the arena facing different walls in each session to promote allocentric navigation (right). **(B)** Schema memory task. Animals either performed four (Low information load) or eight (High information load) encoding episodes, with an inter-trial interval of 20 min. In each episode, the rats explored different pairs of identical objects for 3/5 min, which were arranged according to a spatial rule across trials, i.e., one location was always (red zone) and two locations were only partially occupied (blue zones). Schema recognition memory (of the spatial rule) was tested 24 h later (dashed arrows). For this, objects from the first encoding episode were moved, so that both occupied locations different from encoding. The location of one object (red arrows) thus violated the spatial rule. Schema memory is assessed based on the increased exploration time the animal devotes to the object violating the spatial rule in comparison to the time spent exploring the other object. **(C)** Episodic memory task. Animals also performed on either four (Low information load) or eight (High information load) encoding episodes with different pairs of identical objects on each episode. But, objects were arranged without a spatial rule, with all locations equally often occupied by an object across episodes. Episodic memory for the last encoding episode was tested 24 h later (dashed arrows) with the objects from this episode arranged such that one object was moved to a novel position (displaced object, red arrows), while the other remained at the familiar location (stationary object). Episodic recognition memory was assessed based on the increased exploration time the animal devoted to the displaced object in comparison to the time spent exploring the stationary object.

Twenty-four hours after the last habituation session, the rats were again brought to the test room for the encoding phase of the elaborated version of the OPR task. The encoding phase comprised either eight (high information load) or four (low information load) consecutive encoding episodes separated by an inter-trial interval of 20 min. In each trial, a different pair of identical objects were placed in two out of eight possible locations in the arena, ensuring that objects were equally distant to the arena walls (10 cm from the bottom of the objects, Figure 1). For the schema version of the task, one location inside the arena was occupied by an object during all encoding episodes, while two other locations were occupied by an object every second episode. Thus, a spatial rule existed across encoding episodes such that one location was always occupied by an object, whereas two locations were only partially occupied across episodes. Accordingly, the spatial rule was sufficiently presented only after the animal had completed at least two encoding episodes. For the episodic memory version of the task, no such spatial rule was present across the encoding episodes. Instead, the object pairs were placed semi-randomly in two out of eight possible locations in the arena, with the constraint that all possible locations were occupied equally often across episodes (Figure 1C).

For all experiments, animals entered the open field facing a different wall of the arena at each encoding episode to promote the formation of an allocentric spatial representation. The duration of each encoding episode was 5 min. For practical reasons, in subgroups of six animals in each the low and high information load conditions, encoding duration was reduced to 3 min. The explorative behavior of these animals did not differ from those with 5-min episodes, *p* > 0.10 for all relevant parameters. During the inter-trial interval, animals were kept in their home cage and after completion of the encoding phase, animals were brought back into the animal facility.

Twenty-four hours after the encoding phase, animals were brought back to the test room and tested for either memory of the spatial rule or episodic memory of the last encoding episode. In the schema version of the task, the object pair used in the first encoding episode was again placed in the arena. However, this time the object that had been placed at the always occupied location during encoding was moved to a location that had never been occupied during the encoding phase, while the other object was moved to the location that was partially occupied during the encoding phase but, had not been occupied during the first encoding episode. Thus, both objects were moved to a location different from that during the encoding episode, but only the placement of one object violated the spatial rule (i.e., that one location is always occupied by an object) enabling the separate assessment of schema memory.

The episodic version of the task (not comprising a spatial rule at encoding) should provide a separate measure of episodic memory unbiased by any schema memory formation. For testing episodic memory, the object pair of the last encoding episode was again placed in the arena and, like in the classical OPR task, one of the objects was moved to a different location while the other (stationary) object remained at the same location as during encoding. We focused on the last encoding episode to exclude the effects of (retroactive) interference. Animals from both high and low information load groups were subjected to the same procedure during the test session. The duration of the test trial was 5 min for all groups.

Ten animals were randomly assigned to each experimental group, i.e., low information load/schema memory, high information load/schema memory, low information load/episodic memory, and high information load/episodic memory, according to a between-groups design. All experiments were carried out between 8:00 a.m. and 14:00 p.m. Locations in which objects were placed and the type of objects were randomized across encoding and test phases.

To assess memory performance, exploratory behavior directed towards the objects during the encoding and test trials was manually scored after the completion of all experiments using tracking software (ANY-maze, Stoelting Europe, Dublin, Ireland). Object exploration was defined as the rat being within 1 cm of an object, directing its nose towards the object, and engaging in active exploration behaviors such as sniffing. Leaning on the object without sniffing close to the object (>1 cm) was not counted as object exploration behavior. All scoring was done by the same experienced experimenter, who was blinded to the experimental condition. To assess memory retrieval for the spatial rule (schema memory) or object-place recognition memory (episodic memory) a discrimination ratio was calculated according to the general formula:


$$\frac{object\exp lorationtimeatnovellocation-object\exp lorationtimeatfamiliarlocation}{object\exp lorationtimeatnovellocation+object\exp lorationtimeatfamiliarlocation}$$

Novel location refers to the object at a previously never occupied location in the schema memory test and to the displaced object in the episodic memory test. A positive discrimination ratio indicates memory for the spatial rule or for the stationary object, respectively, whereas a value of zero indicates no exploration preference.

### Data Reduction and Statistical Analyses

To assess indicators of motivation and locomotion, the total object exploration time and distance traveled during encoding and test phases were extracted from the videos. This data was then further analyzed using statistical software (R, R Core Developer team). Data from individual rats were discarded, when animals exhibited consistently low exploration times during the encoding phase, specifically when rats spent <1 s exploring both objects in more than 50% of the episodes. This resulted in four rats being discarded from the dataset and a total number of 36 rats (low information load/schema memory *n* = 9, high information load/schema memory *n* = 9, low information load/episodic memory *n* = 9, high information load/episodic memory *n* = 9) included in the final analyses.

Discrimination ratios from the test phase were calculated separately for each minute and the total 5-min duration. For statistical analyses, a mixed linear model was fitted using the lm4 package (Bates et al., 2015) with individual rats as random effect (random intercept only) and the fixed effects Information Load (High vs. Low), Task (Schema vs. Episodic) and Minute (1st vs. 5th minute of test trial):


DR∼(InfoLoad∗task∗Minute)+(1|animal)

where DR indicated the discrimination ratio over the 5-min test interval. The significance of factors was assessed by removing the respective factor or interaction of two factors step by step from the model and comparing the modified models with the original using likelihood-ratio tests. In addition, control parameters including total distance traveled and total object exploration time during encoding and test trials were analyzed using the same approach. For comparisons, two-sided Welch *t*-tests were computed. Correlational analyses were based on Spearman correlation coefficients to account for the low number of animals and were compared using both, Fisher’s z and Zou’s confidence intervals (level of confidence: 0.95) as implemented in the corcor toolbox (Diedenhofen and Musch, 2015). For all analyses a *p* < 0.05 was considered significant. Results are reported as the means ± SEM.

## Results

**Figure 2:**
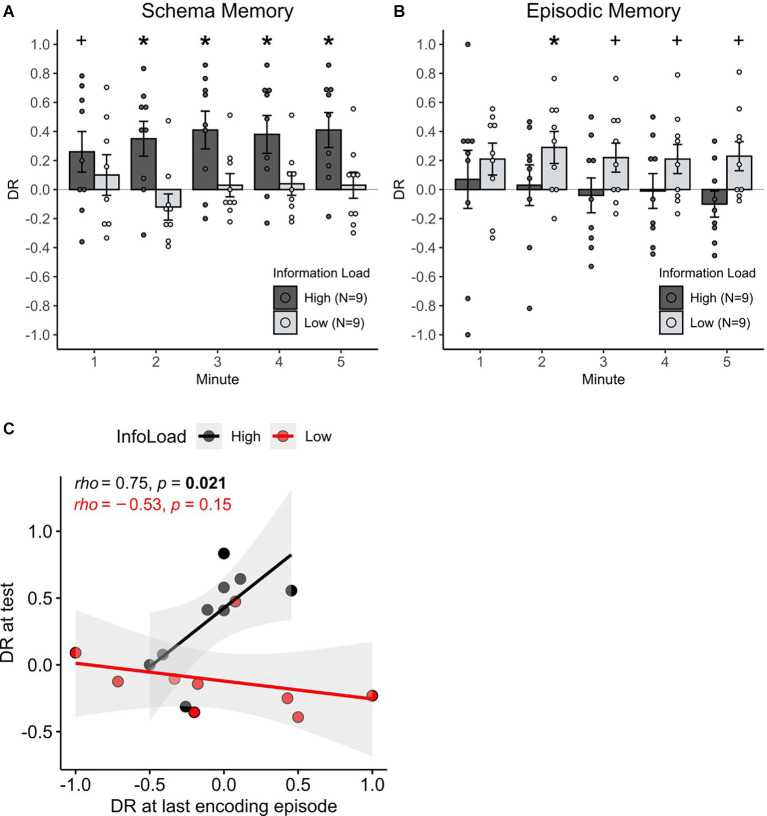
The effect of information load on episodic and schema memory formation. **(A)** Mean + S.E.M. cumulative discrimination ratios during the 5-min test phase for the schema version of the task (*n* = 18), and **(B)** for the episodic version of the task (*n* = 18). Red asterisks indicate significant (above chance) memory per min (*p < 0.05, ^+^*p* < 0.1). Schema memory for the spatial rule was expressed only in the high, but not in the low information load condition. Episodic memory was transiently expressed only in the low information load condition (2nd min). Significance for the information load (high, low) × memory type (schema, episodic) interaction (*p* < 0.001) points to an effect of episodic vs. schema memory in opposite directions. **(C)** Correlations of the discriminatory exploration towards the partially occupied location (DR > 0) at the last encoding episode in the high and low information load condition of the schema version of the task and the respective memory performance at the test. Ratios are taken from the first 2 min of the encoding and test episodes since memory expression was clearly present in that minute. Correlations significantly differ between the groups (*p* < 0.01). Note the significant positive correlation for the high information load condition suggests an emergent schema representation during the last encoding episode is predictive for schema memory recall at the test.

At the test phase, 24 h after encoding, the rats exhibited significant schema memory only in the high information load condition (χ^2^_(1)_ = 5.99, *p* = 0.014, for the difference in discrimination ratios between high and low information load during schema memory testing, Figure 2A). In the high information load condition, schema memory performance above chance manifested itself after the first minute of the test phase (all *p* < 0.02) and approached significance already in the first minute (*t*_(8)_ = 1.89, *p* = 0.09). When animals performed only four learning trials in the low information condition, no memory above chance was found (all *p* > 0.2). Correlational analysis revealed that only in the high information load condition a high preference for the partially occupied location during the last encoding episode was predictive of a higher memory performance at the test (*rho* = 0.75, *p* = 0.021), but not in the low information load condition (*rho* = 0.078, *p* = 0.76, *z* = 2.69, *p* = 0.007 for difference between correlations, Figure 2C). These findings indicate that only in the high information load animals were able to form and retrieve schema memory for the spatial rule that was present during the encoding trials.

In contrast, on the episodic memory test, the rats exhibited an object-place memory for individual encoding episodes that was above chance, only in the low information load condition. Respective discrimination ratios were significant in the 2nd min of the test phase (*t*_(8)_ = 2.70, *p* = 0.02, Figure 2B) and approached significance in minutes 3–5 (all *p* < 0.09). Animals in the high information load condition did never exhibit discrimination ratios above chance (all *p* > 0.2). The difference between the low and high information load conditions across all minutes did not reach significance, however (χ^2^_(1)_ = 2.49, *p* = 0.113). Overall, these findings hint towards a modulating role of information load for episodic memory, with this effect, however, being weaker than on the formation of schema memory.

To address whether the effect of information load during encoding on episodic vs. schema memory formation acts in opposing directions, the discrimination ratios across all experimental groups were compared. Evidence for such an opposing effect was indeed present, as the expression of recognition memory was dependent on an interaction between information load and the type of memory assessed (χ^2^_(1)_ = 8.04, *p* = 0.004 for Information load × Schema/Episodic memory interaction).

**Figure 3:**
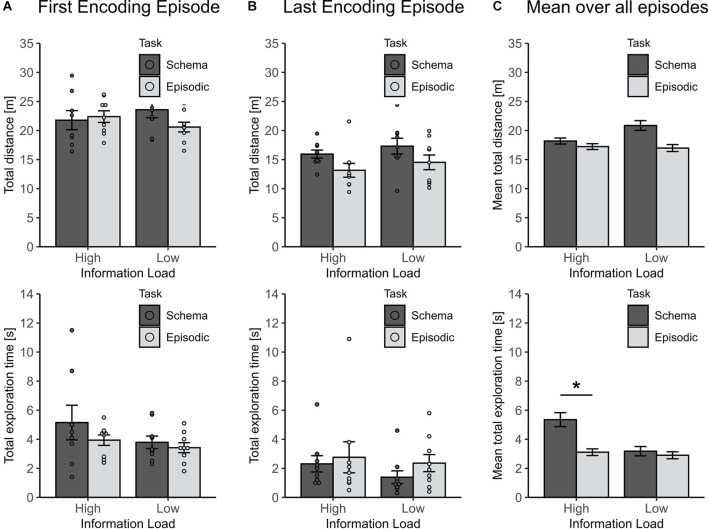
Locomotion parameters for encoding episodes to assess unspecific motivational differences between experimental groups. **(A)** For the first encoding episode traveled distance and total exploration time were comparable across conditions (all *p* > 0.1). **(B)** For the last encoding episode (either the fourth or eighth in the low or high information load condition, respectively) the traveled distance decreased more in the high than in low-information load condition (*p* = 0.019), whereas the total exploration time remained comparable across groups (*p* > 0.3). **(C)** Mean distance traveled over all encoding episodes was lower in the high than in the low information load condition (*p* = 0.018). Unexpectedly, the mean total exploration time across all episodes depended on an interaction of Information Load and Type of Memory (*p* = 0.037) with this effect largely driven by higher mean exploration time in the High Load/Schema condition (lower panel). Statistically controlling for this effect did not change results for retrieval (see text). *p < 0.05.

To exclude that the observed effects resulted from unspecific motivational differences between the groups during encoding, the traveled distance and total object exploration time across all encoding episodes, and during the first and last episode were compared (To include all animals, this was done for the first 3 min of each episode). For the first encoding episode, neither the traveled distance nor the total object exploration time differed between groups (all *p* > 0.1), ruling out any unspecific differences (Figure 3A). For the last encoding episode (i.e., the fourth and eighth, respectively) the traveled distance decreased more in the high than in the low information load condition (χ^2^_(1)_ = 6.04, *p* = 0.019), while the total object exploration time remained comparable across groups (all *p* > 0.3, see Figure 3B). Indeed, the decrease in locomotion is plausible suggesting a higher level of habituation for animals that spent a greater number of episodes in the arena. In line with this finding, also the mean traveled distance across all encoding episodes was revealed to be lower in the high than low information load condition (χ^2^_(1)_ = 5.57, *p* = 0.018, Figure 3C, left panel).

Unexpectedly, mean total object exploration time across all encoding episodes depended on an interaction between information load and type of task (χ^2^_(1)_ = 4.31, *p* = 0.037) which was largely driven by longer mean exploration durations in animals of the high information/schema memory group (Figure 3C, lower panel). While this effect is difficult to explain, we excluded the possibility that the longer exploration durations in this group contaminated the observed effects of episodic vs. schema memory by running a separate mixed model analysis that controlled for the mean exploration time at sampling. This analysis confirmed the initial finding of the significant effect of information load on the formation of episodic vs. schema memory (*p* = 0.00017, for respective Information load × Schema/Episodic memory interaction).

## Discussion

The abstraction of gist information from multiple experiences into schema memory and the formation of detailed episodic memory from individual experiences serve different functions for the mammalian memory system (Wang and Morris, 2010). Whether these two processes can occur in parallel or compete with each other, is unknown. On the one hand, previous studies have demonstrated that the consolidation of declarative memories is impaired if the information load during learning is too large (Feld et al., 2016; Kolibius et al., 2021). On the other hand, it is known that schema learning benefits from higher loads of information, from which gist information can be extracted (Tse et al., 2007; Wang and Morris, 2010). Against this backdrop, the results of this study suggest that the formation of detailed episodic memory and generalized schema memory for a rule, indeed, depends on information load during learning in opposite direction. Animals were able to recognize a violation of the spatial rule present during encoding 24 h earlier only when exposed to eight episodes, but not to just four episodes during encoding. In contrast, episodic details of the last encoding episode were only retained after four but not eight consecutive encoding episodes.

The effect of information load on the formation of declarative memory has been previously tested in humans using a word-pair retention task (Feld et al., 2016; Kolibius et al., 2021). Learning large lists of word pairs, i.e., a high information load during learning, did not benefit from sleep-dependent memory consolidation processes, in contrast to learning shorter word pair lists. To explain this difference, it was proposed that active systems memory consolidation, a process that likely operates during sleep, is capacity-limited (Kolibius et al., 2021). We did not systematically assess to what extent our rats slept after the encoding phase, however, all experiments were carried out in the morning between 8:00 a.m. and 2:00 p.m. when sleep pressure in rodents is high (Van Twyver, 1969). Hence, post-encoding sleep might have been a factor significantly contributing to the present results in which the rats expressed episodic memory, although only transiently, in the low but not in the high information condition. Alternatively, decreased episodic memory formation with high loads of information encoded may be viewed as a consequence of increased non-specific interference, independent of the occurrence of sleep after encoding (Wixted, 2004; Yonelinas et al., 2019). Indeed, it has been suggested that familiarity-based memories are especially sensitive to interference (Sadeh et al., 2016), which is of importance as the memory test in the object-location preference task relies on familiarity. We aimed to reduce the interference in the task by testing episodic memory for the last encoding episode. However, while this strategy is sufficient for reducing retroactive interference, it cannot rule out proactive interference, i.e., the process in which previously learned information impairs the learning of new information (Brawn et al., 2018). In this view, the effect of information load on episodic memory might be mediated by proactive interference created by the task.

The opposite dependencies of schema vs. episodic memory performance on information load during encoding in our study hints toward a competitive relationship between the formation of these types of memory. A factor that could explain this competitive relationship might be the generally limited capacity of hippocampal networks for processing episodic memory information. In adapting the OPR task to the purpose of the present study, each encoding episode of the task used a unique object-location pairing. Accordingly, in the episodic version of the task, the information load scaled linearly with each additional encoding episode. It is likely that, with an increasing number of unique episodes, the capacity for episodic memory processing in hippocampal networks is at some point surpassed, and the information is forgotten, be it during sleep to prevent episodic memories from undergoing active systems consolidation, or during wake to mutually weaken episodic memories through interference. As to the episodic memory test, this scenario would explain the absence of episodic memory in the high information load, but it might also partly account for the low information load condition, where the expressed episodic memory for the last encoding episode was rather weak and only transient (i.e., in the 2nd minute). Conversely, in the schema version of the task each encoding episode adds information about the spatial rule and information density for the rule, therefore, decreases over an increasing number of encoding episodes. At the level of hippocampal networks, this decrease in information density could be associated with an increased representational overlap between the individual encoding episodes that eventually facilitates abstraction of a more general schema memory for the spatial rule (Lewis and Durrant, 2011). If so, the postulated capacity limit should not interfere with the test of memory for the rule. Indeed, our data together with the inferred increase in representational overlap possibly facilitating schema memory formation, is also well in line with findings indicating that more repetitions are beneficial for the formation of schema memory, whereas a low information load at learning might not be sufficient (Tse et al., 2007; McKenzie et al., 2014).

The conclusion that the opposite effects of information load on schema vs. episodic memory reflect a competitive relationship between these memories, may be questioned based on the fact that, rather than probing both kinds of memory with the same stimulus materials, task stimuli differed between the schema and episodic versions of the task, with only the former comprising the spatial rule across encoding episodes. However, using the same behavioral readout (i.e., exploration of novelty) for probing episodic and schema memory in the used adaptation of the OPR-based task, it is basically impossible to independently assess both kinds of memory on an identical set of stimuli during encoding, simply because a set of encoding episodes that allows for abstracting a spatial rule across episodes, necessarily allows for the simultaneous formation of episodic memories for the individual encoding episodes. Specifically, this means that the schema version of the task cannot be used to independently assess episodic memory. In principle, an assessment of memory for an individual encoding episode would require that at the test, only one of the objects of the respective episode is displaced such that the original spatial configuration of this episode changes but the rule across episodes is continued (i.e., one object stays at the always occupied location and the other switches to the formerly not occupied partially occupied location). Such test configuration, however, does not allow for a valid test of pure episodic memory, as it could well be biased by the continuation of the rule and the resulting rule knowledge (making the respective episodic change in the location of the object appearing less novel). Moreover, animals forming memory in a cumulative manner across multiple episodes have been found to prefer exploring the less often occupied location over the always occupied location, independently of whether or not the less often occupied location violates an emergent rule (Genzel et al., 2019). Note, for testing schema memory separately from episodic memory, we, therefore, displaced both objects of the respective episode to another location (one violating the rule and the other deviating from the spatial configuration of this particular episode). This test configuration is expected to elicit parallel exploration driven by episodic memory and exploration driven by schema memory, but only an activated schema memory would drive a differential exploration towards the object in the novel location, i.e., the one violating the spatial rule, as it was found in the high information condition.

However, despite the proposed competitive relationship between episodic and schema memory, based on the present findings, it is impossible to rule out alternative explanations. Since episodic memory could not be assessed in the schema version of the task, one might alternatively explain the effect of information load on schema memory based on the occurrence of retro- and proactive interference across episodes (Wixted, 2004). For instance, if animals in the low load condition of the schema memory task had formed, at the test phase, both schema memory as well as episodic memory for the first encoding episode, both objects on the never-occupied (rule-violating) location and on the partially occupied (rule-continuing) location, would represent “unfamiliar” locations, and the zero-discrimination found in this condition would not indicate the absence of schema memory, but the sole presence of episodic memory or the joint presence of episodic and schema memory that cancel each other out. In this scenario, an increase of interference as a result of higher information load results in a weaker episodic memory and, thus, a clearer schema memory expression. In order to test whether or not schema memory is actually formed already after four encoding episodes (i.e., in the low load condition), our schema memory task that was based on a classical OPR-task design would need further modification. For example, a third object could be added to each episode such that there is repeating (i.e., schema relevant) information and unique (i.e., episodic) information available that can be contrasted in a memory test phase. Such modification clearly separating shared and item-unique information would make the task similar to the Satellite task used in humans (Schapiro et al., 2017). However, it still would not solve the problem of interpreting a zero-discrimination ratio indicating either the absence of memory or the presence of both episodic and schema memory that cancel each other out, rendering appropriate control conditions vital to the interpretation of behavioral effects.

To explain our behavioral results, one might also refer to the concept of habituation, i.e., after eight encoding episodes, rats at the test in the schema version of the task preferentially explored the object that violated the rule because they were more habituated to the presence of an object in the partially occupied locations. However, the process of habituation when considered as a learning process across episodes with differing stimulus configurations does not exclude processes of schema memory formation, but would rather explain the changes in behavior at a different epistemological level. Note, that the objects used in the different episodes were clearly discriminable for the rats and, interestingly, a supplementary analysis revealed no clear signs of habituation across encoding episodes, in terms of a decreased exploration toward the always occupied location across episodes (see Supplementary Figure 3 for respective learning curves).

The formation of schema memory is often thought to be a slow process that evolves during systems consolidations over longer time intervals, i.e., days and even weeks (Walker and Stickgold, 2010; Lewis and Durrant, 2011; Dudai et al., 2015), although there is no minimum time required to form a schema. In this study schema memory for a spatial rule was formed in ~3 h and retrieved within 24 h in the high information load condition. Does this quick timescale contradict the concept of schema memory? Previous studies indicate that the speed at which schema memories are formed essentially depends on the presence of pre-existing knowledge into which respective information can be readily integrated (Gilboa and Marlatte, 2017). Even schema memories that derive from reoccurring patterns over multiple episodes may rather rapidly form when the relevant information can be readily assimilated into pre-existing representations (Tse et al., 2007). Pre-existing knowledge may have also accelerated schema memory formation in the present experiment: The rats were thoroughly habituated to the arena before the experiments to develop an allocentric spatial map of the arena environment. Also, experiences of the general procedures including the habituation to the experimenter, the rat’s journey back and forth to the experimental room, etc. might have formed memories representing an abstract knowledge about the commonalities across days. These and related representations might have served as a scaffold facilitating the formation of schema memory arising in the same environmental context, especially under high information load conditions. An interesting question not addressed here is whether in low information load conditions the abstraction of a schema memory for the spatial rule would unfold with longer periods of active consolidation (Nader et al., 2000; Binder et al., 2012; Dudai, 2012).

Overall, the present results indicate that the amount of encoded information impacts, in opposite directions, the formation of schema and episodic memory. A factor contributing to this effect might be the limited capacity of hippocampal networks for processing memory information, enforcing representational overlap to augment schema formation in conditions of high information load, whereas episodic memory can freely form in conditions of low information load. However, modifications to the task design are needed to directly assess the proposed competitive relationship. Our study demonstrates in principle that OPR-based tasks offer a promising approach to the combined study of episodic and schema memory dynamics in rodents.

## Data Availability Statement

The raw data supporting the conclusions of this article will be made available by the authors, without undue reservation.

## Ethics Statement

The animal study was reviewed and approved by Baden-Wuerttemberg state authorities.

## Author Contributions

MC and MH carried out the experiments. MH performed all the analyses. MC, MI, and JB conceived the original experiment. All authors contributed to the article and approved the submitted version.

## Conflict of Interest

The authors declare that the research was conducted in the absence of any commercial or financial relationships that could be construed as a potential conflict of interest.

## Publisher’s Note

All claims expressed in this article are solely those of the authors and do not necessarily represent those of their affiliated organizations, or those of the publisher, the editors and the reviewers. Any product that may be evaluated in this article, or claim that may be made by its manufacturer, is not guaranteed or endorsed by the publisher.
