# Supplementary material for: Effects of Information Load on Schema and Episodic Memory Formation
Source: Front Behav Neurosci. 2022 Jul 11;16:923713. doi: 10.3389/fnbeh.2022.923713 (PMC9315445; doi:10.3389/fnbeh.2022.923713)
Supplement: Supplementary file 1 [file Data_Sheet_1.pdf]

Supplementary Material

1 Supplementary Figures

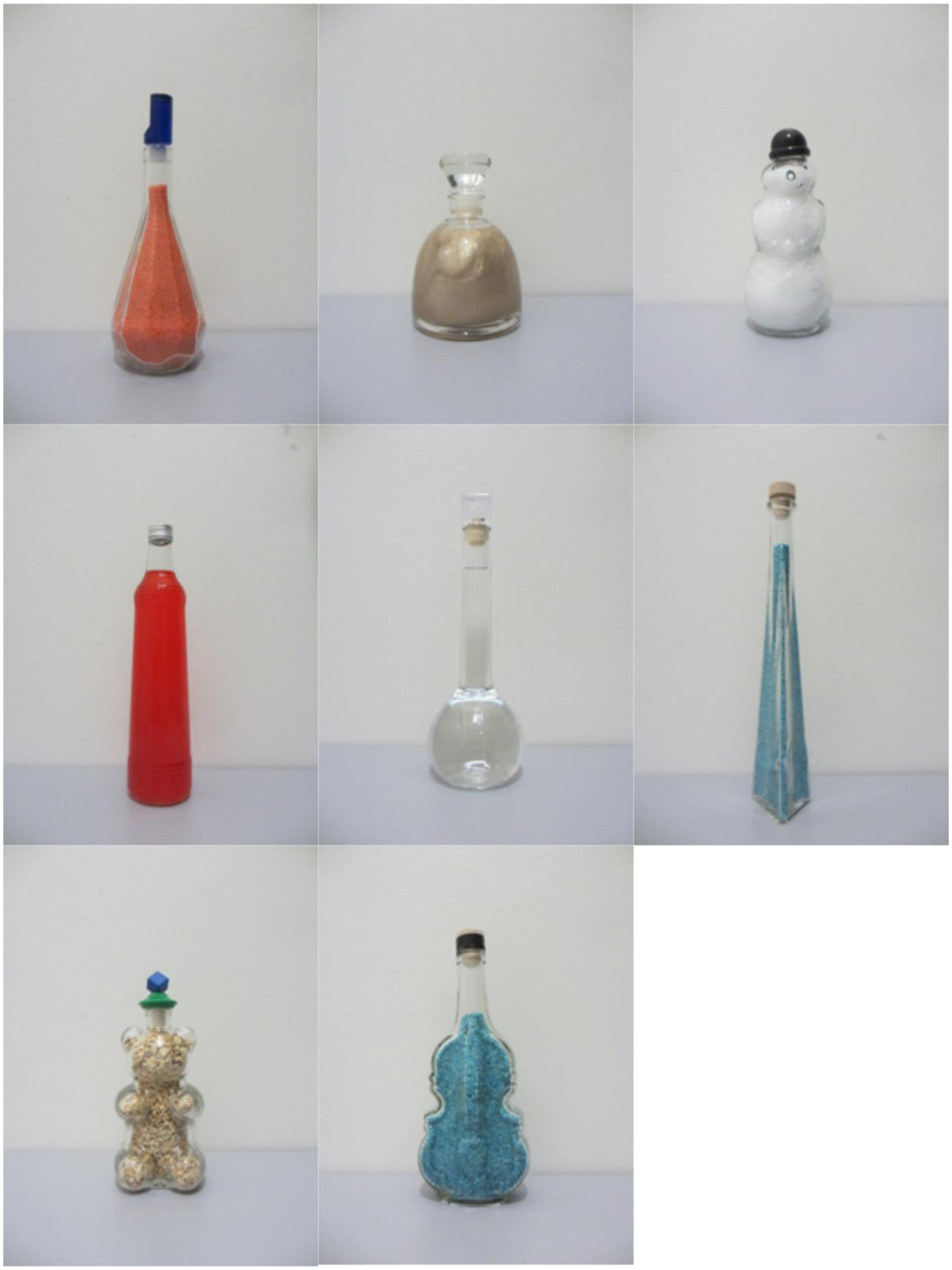

Supplementary Figure 1. Eight glass objects of different shapes and sizes (height 15-30 cm, bottom diameter 7-12 cm), filled with sand of different colors, were used in the experiments

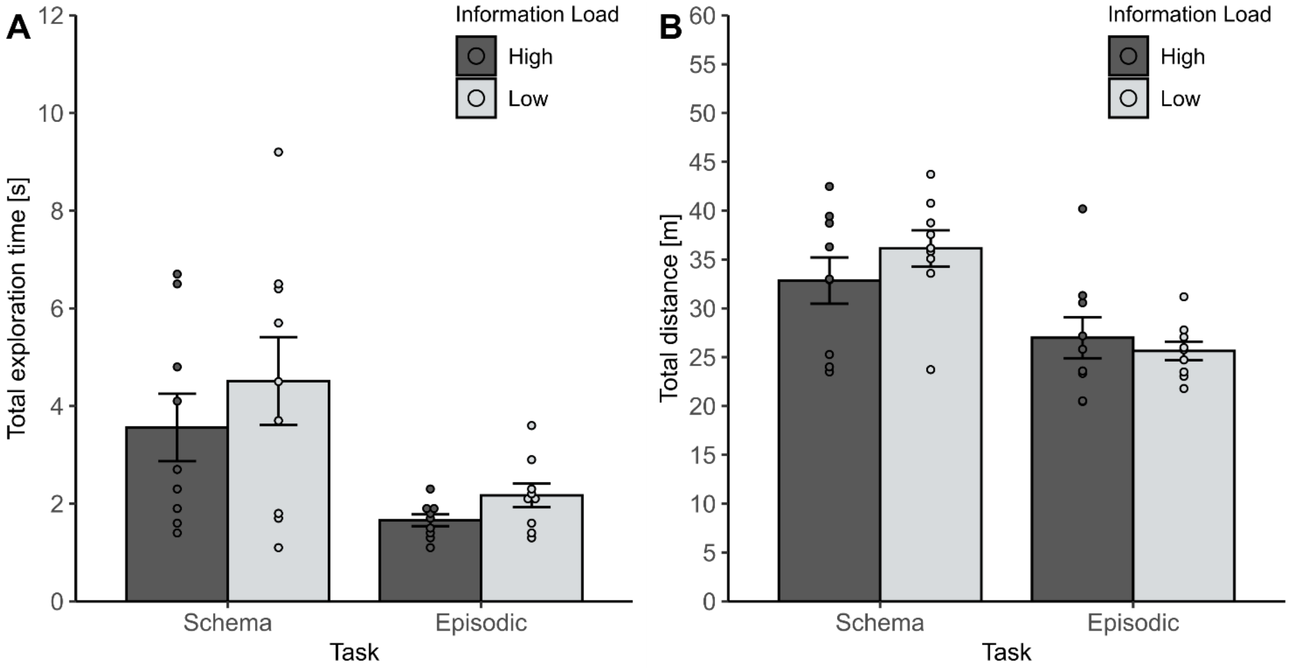

Supplementary Figure 2. Control parameters, **(A)** Total exploration time and **(B)** Total distance travelled during the 5-min duration of the test phase. Importantly, total exploration time and travelled distance were comparable between high and low information load conditions in both the schema and episodic memory tests ($p > 0.2$, for all comparisons). Unexpectedly, both parameters were generally increased during schema memory testing compared with episodic memory testing ($\chi2[1] = 13.6$ and 18.2, respectively, $p < 0.001$) which is difficult to explain. It possibly reflects increased non-specific arousal accompanying the violation of the rule, i.e., the detection that something has changed, in the absence of a specific memory for the object location violating the rule.

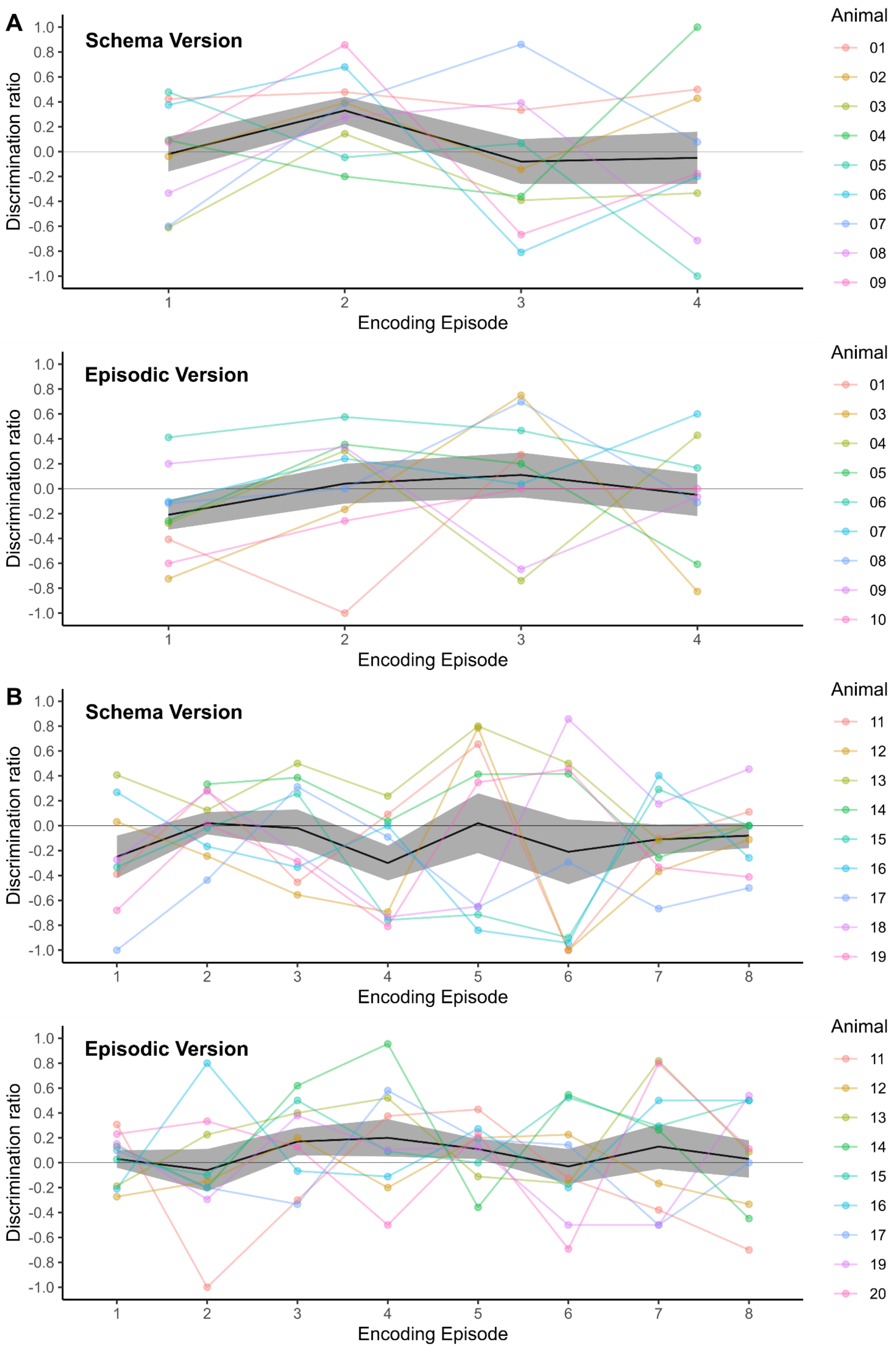

Supplementary Figure 3. Change of discrimination ratios (DR) across encoding episodes for each animal and the Mean ± SEM, for the (A) low load condition (i.e. 4 episodes) and (B) high load condition (i.e. 8 episodes). For the schema version of the task (upper panels) a positive DR reflects a

preferential exploration towards the object presented at the partially occupied location, and a DR of zero no preferential exploration. A systematic change across encoding episodes in the schema version of the task would, thus, be indicative for emergent schema learning, i.e., when animals start to prefer the partially occupied location over the always occupied location. For the episodic version of the task (lower panels) object presentation did not follow a spatial rule across episodes and, therefore, no learning is expected. There is no evidence for any emerging exploratory preference for the schema version or episodic version of the task.
